# Building Disease-Specific Drug-Protein Connectivity Maps from Molecular Interaction Networks and PubMed Abstracts

**DOI:** 10.1371/journal.pcbi.1000450

**Published:** 2009-07-31

**Authors:** Jiao Li, Xiaoyan Zhu, Jake Yue Chen

**Affiliations:** 1 State Key Laboratory of Intelligent Technology and Systems, Tsinghua National Laboratory for Information Science and Technology, Department of Computer Science and Technology, Tsinghua University, Beijing, China; 2 School of Informatics, Indiana University, Indianapolis, Indiana, United States of America; 3 Department of Computer & Information Science, School of Science, Purdue University, Indianapolis, Indiana, United States of America; 4 Indiana Center for Systems Biology and Personalized Medicine, Indiana University–Purdue University, Indianapolis, Indiana, United States of America; University of Chicago, United States of America

## Abstract

The recently proposed concept of molecular connectivity maps enables researchers to integrate experimental measurements of genes, proteins, metabolites, and drug compounds under similar biological conditions. The study of these maps provides opportunities for future toxicogenomics and drug discovery applications. We developed a computational framework to build disease-specific drug-protein connectivity maps. We integrated gene/protein and drug connectivity information based on protein interaction networks and literature mining, without requiring gene expression profile information derived from drug perturbation experiments on disease samples. We described the development and application of this computational framework using Alzheimer's Disease (AD) as a primary example in three steps. First, molecular interaction networks were incorporated to reduce bias and improve relevance of AD seed proteins. Second, PubMed abstracts were used to retrieve enriched drug terms that are indirectly associated with AD through molecular mechanistic studies. Third and lastly, a comprehensive AD connectivity map was created by relating enriched drugs and related proteins in literature. We showed that this molecular connectivity map development approach outperformed both curated drug target databases and conventional information retrieval systems. Our initial explorations of the AD connectivity map yielded a new hypothesis that diltiazem and quinidine may be investigated as candidate drugs for AD treatment. Molecular connectivity maps derived computationally can help study molecular signature differences between different classes of drugs in specific disease contexts. To achieve overall good data coverage and quality, a series of statistical methods have been developed to overcome high levels of data noise in biological networks and literature mining results. Further development of computational molecular connectivity maps to cover major disease areas will likely set up a new model for drug development, in which therapeutic/toxicological profiles of candidate drugs can be checked computationally before costly clinical trials begin.

## Author Summary

Molecular connectivity maps between drugs and a wide range of bio-molecular entities can help researchers to study and compare the molecular therapeutic/toxicological profiles of many candidate drugs. Recent studies in this area have focused on linking drug molecules and genes in specific disease contexts using drug-perturbed gene expression experiments, which can be costly and time-consuming to derive. In this paper, we developed a computational framework to build disease-specific drug-protein connectivity maps, by mining molecular interaction networks and PubMed abstracts. Using Alzheimer's Disease (AD) as a case study, we described how drug-protein molecular connectivity maps can be constructed to overcome data coverage and noise issues inherent in automatically extracted results. We showed that this new approach outperformed both curated drug target databases and conventional text mining systems in retrieving disease-related drugs, with an overall balanced performance of sensitivity, specificity, and positive predictive values. The AD molecular connectivity map contained novel information on AD-related genes/proteins, AD candidate drugs, and protein therapeutic/toxicological profiles of all the AD candidate drugs. Bi-clustering of the molecular connectivity map revealed interesting patterns of functionally similar proteins and drugs, therefore creating new opportunities for future drug development applications.

## Introduction

The concept of *molecular connectivity maps* is gaining popularity in systems biology [1]. Massive amounts of genomics and functional genomics information, including genome-wide genetic variations, epigenetic modifications, mRNA expression profiles, protein expression profiles, protein post-translational modifications, and metabolic profile changes in cells, have been generated [2]. While there is steady progress in managing and interpreting data for each type of measurement individually, it remains uncertain yet rewarding how to develop unified models—even descriptive ones to begin with—to integrate signals from genomic-scale measurements of different molecular entities under similar biological conditions. In modern drug discovery, for example, the expression level of genes or proteins that change in response to different drug compound perturbations, or “drug- gene/protein association profiles”, are believed to provide valuable prescience on the drug's molecular potential therapeutic and toxicological profiles prior to clinical trials. Here, the concept of studying “inter-class” molecular associations is quite different from that of “intra-class” molecular associations such as gene-gene interactions, drug-drug interactions, or protein-protein interactions [3]. For example, differential gene expression profiles based on DNA microarrays were used in an *inter-class* molecular study to link several genes, *efpA*, *fadE23*, *fadE24*, *ahpC*, to the toxic response of anti-tuberculous drug *isoniazid* [4]. In this study, an *inter-class* drug-gene molecular association profile was established between the drug *isoniazid* and several tuberculosis-related genes. Generalizing from the concept of gene-drug molecular connectivity profiles built from a few drugs or genes, we refer to the comprehensive inter-class molecular associations in a given biological context as a *molecular connectivity map*. Molecular connectivity maps may be developed between drugs and a wide range of bio-molecular entities such as genes, microRNAs, proteins, and metabolites for a variety of disease areas. A high-quality molecular connectivity map can enable researchers to compare the molecular therapeutic/toxicological profiles of many candidate drugs or drug target genes/proteins, therefore improving the chance of developing high-quality drugs and reducing drug development time.

Several large-scale molecular connectivity mapping projects are currently under way. Lamb *et al.* recently established a systematic approach to build connectivity maps using gene-expression profile information as the common vocabulary that connects small molecules, genes, and diseases [5]. These connectivity maps consist of a reference collection of gene-expression profiles from cultured human cells treated with bioactive small molecules. The map data also come with pattern-matching software to help researchers query these maps [1]. Butte *et al.* proposed a different strategy, using the UMLS (Unified Medical Language System) ontology and publically available gene expression data to associate a broad spectrum of “vantage points”—biologically significant terms used in phenotypic, disease, environmental and experimental contexts—with genes [6]. While both approaches open up new opportunities that make it possible to observe molecular connectivity profiles in parallel, the coverage and quality of these connectivity maps raise doubts. Lamb *et al.*'s approach relies on systematic screening of each known chemical compound against cell lines simulating each biological condition to derive gene expression profile changes—a costly and time-consuming experimental process that will take many years and a huge budget before sufficient data coverage can be achieved for practical use. The approach by Butte *et al.* relies heavily on integrating available gene expression data from different laboratories running different experimental platforms on different biological samples—sometimes producing incompatible results that require thorough in-depth experimental validations or knowledge curation.

Is it possible to build high-quality low-cost molecular connectivity maps? To answer this question, one must resort to the vast amount of biomedical literature and emerging biomedical literature mining techniques. Recent advances in biomedical information retrieval [7], gene/protein entity identification [8], information extraction [9], text clustering and classification [10], and the integration of structured and textual data [11], have made it practical to perform knowledge discovery in primary biomedical literature [12]. There are quite a few successful examples. FACTA is a biomedical literature search engine for identifying biomedical concepts (e.g., disease, gene/protein, chemical compounds) from PubMed abstracts [13]. G2D is a tool for inferring logical chains of connections from disease names and ranked genes on the basis of a score that represents their likelihood of being associated with the query disease [14]. Tiffin *et al*. identified co-occurring disease names and tissue names in PubMed abstracts, and linked the tissues to candidate disease genes [15]. Srinivasan *et al.* developed a method to explore implicit relationships between pharmacology substances and diseases [16]. Given disease names and user-specified terms, these biomedical literature-mining techniques were capable of prioritizing terms (*e.g.*, genes, tissues, and substances etc.) with potential roles in the diseases. It is quite tempting to conclude that molecular connectivity maps could be built theoretically by searching, collecting, and “triangulating” disease-gene, disease-drug, and gene-drug term co-occurrences, using existing literature mining methods. The real challenge, however, is how to achieve satisfactory sensitivity and specificity from diseases to drugs, while enabling the discovery of *novel* therapeutic applications for *known* drugs. An approach that reports only significant associations among protein, drug, and disease terms co-cited in the same article would be undesirable, because there would be no new knowledge connections between molecules and diseases. An approach that either misses many drug entities (low *sensitivity* performance) or assigns unrelated drug entities (low *positive predictive value* performance) would also be depreciated, because human experts, not computers, would have to bear a heavy burden of performing manual knowledge validations.

**Figure 1:**
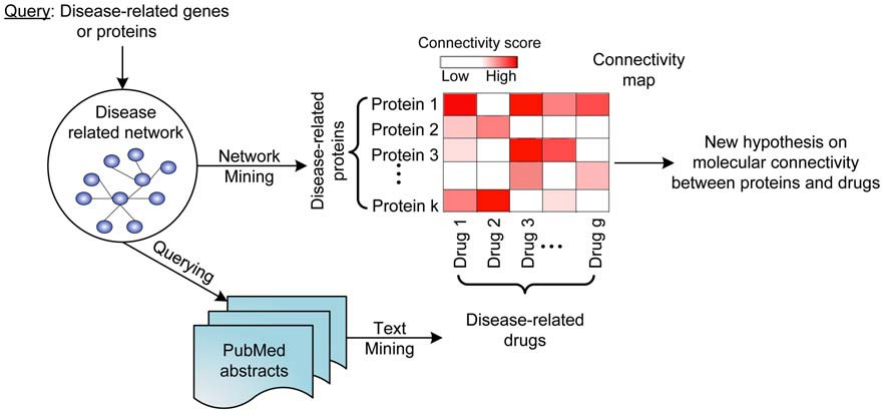
A conceptual paradigm for the development of disease-specific molecular connectivity maps. In this paradigm, molecular interaction data and PubMed abstracts are the primary data sources. Network mining is used to generate disease-related proteins from molecular interactions. Text mining is used to extract disease-related drug terms from PubMed abstracts and to further build drug-protein connectivity map in the disease context.

In this study, we propose a novel computational framework to develop high-coverage disease-specific drug-protein connectivity maps, by applying integrated molecular interaction network mining and text mining techniques (Figure 1). We aim to uncover interesting and non-obvious patterns by relating research publications on genes/proteins, drugs, and disease contexts. The computational paradigm has the following characteristics:

1. It can incorporate the user input of disease-specific seed genes/proteins derived from prior knowledge. Each seed list may be curated by in-house knowledge experts, extracted computationally from large Omics experimental results (*e.g.*, differentially expressed genes from microarray experiments comparing genes between disease samples and normal samples), or retrieved automatically from online curated gene/protein databases for the given disease. While the quality of seeds apparently affects the quality of downstream analysis, these seeds can serve as a starting point and does not need to be complete or optimized.
2. It can automatically improve the quality of initial seed genes/proteins list by expanding and re-ranking them in the functional context by reprioritizing them in disease-related molecular interaction networks. Therefore, the final list of genes/proteins used to build connectivity maps will have heightened relevance to the specific disease context.
3. It can discover drugs implicitly studied across multiple research papers spanning multiple disciplines. This identification of both explicit and implicit drug-protein associations to a disease context is accomplished by the development of sensitive drug term statistics that does not require the disease terms to co-occur in the same abstract.
4. It can summarize the comprehensive knowledge of molecular connectivity data for a given disease context into a 2-D matrix. The 2-D matrix serves as a knowledge map for all proteins and candidate drugs documented in the literature, with each cell in the matrix containing a statistical confidence score indicative of the extent of literature studies involving a specific gene/protein and a drug.

A disease-specific molecular connectivity map built this way is substantially different from one that could be built using conventional literature mining approaches [15]–[18]. In this new approach, not only can the retrieval of disease-related drug from biomedical literature be performed with high sensitivity and specificity, but the opportunity to discover novel therapeutic uses of old drugs also becomes real. A drug may be *re-discovered* in a new disease context, if statistical inference engine that we employs establishes significant links between the drug and the majority of disease-related genes or proteins in thousands of PubMed abstracts. The molecular association profiles for each drug in a particular disease application area can be compared and classified, therefore providing evidence for validating new hypothesis. The potential application of our approach to the identification of new disease therapeutic areas for known drugs—commonly referred to as *drug repurposing*—makes developing molecular connectivity maps particularly interesting.

## Results

**Figure 2:**
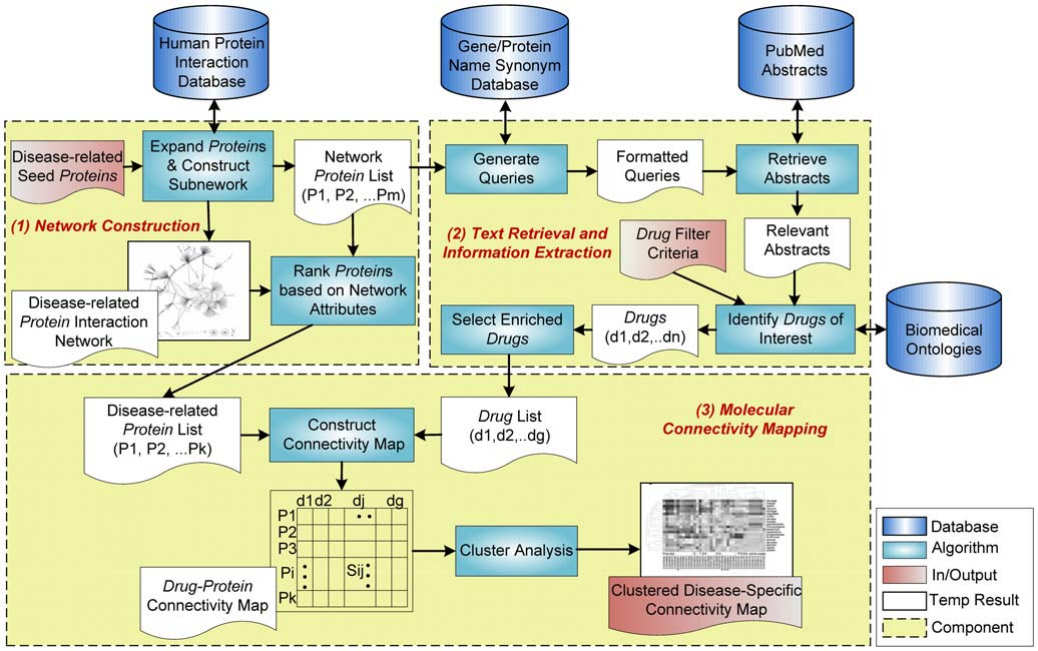
A computational framework for developing molecular connectivity maps in any given disease context. The framework consists of three components: *network construction*, *text retrieval and information extraction*, and *molecular connectivity mapping*. The *network construction* component takes the inputs of disease-specific seed proteins and outputs a disease-related protein interaction network with a ranked list of disease-related proteins. The *text retrieval and information extraction* component takes synonym-expanded disease-related proteins and outputs a list of drug terms enriched in the retrieved collection of PubMed abstracts. The *molecular connectivity mapping* component takes two inputs—disease-related proteins from constructed protein interaction network in the first component, and enriched drug terms in the second component—and outputs a drug-protein connectivity map, in which further knowledge filters and clustering analysis can be applied.

In Figure 2, we show an overview of the computational framework for developing disease-specific molecular connectivity maps. The framework consists of three major components: *network construction*, *text retrieval and information extraction*, and *molecular connectivity mapping*. To help understand how our approach works in action and evaluate its effectiveness, we use Alzheimer's Disease (AD) as a case study. AD is a neurodegenerative disease affecting 4.5 million Americans of mostly over 60 years old and has become an increasingly prevalent disease among senior citizens. The reasons that we choose AD for this study are two-fold: first, the lack of FDA-approved drugs to treat AD today, in spite of decades of research on the disease's molecular mechanisms [19], can make identification of new AD candidate drugs particularly rewarding; second, the wealth of biomedical research articles published for AD studies can make validations of our approach less challenging. We will explain later in Discussion section our approach can be generalized to other disease applications, even those that have not yet been well studied.

### Identification and Refinement of AD-Related Proteins

A key prerequisite to build the molecular connectivity map is to generate both a list of disease-related proteins and a list of disease-related drugs as two attribute dimensions of the protein-drug matrix. The quality of the final map apparently hinges on the overall relevance of proteins and drugs to a particularly disease. In this study, we begin with deriving and optimizing disease-related proteins, because many high-throughput genomics and functional genomics experimental data have made it easy to acquire a preliminary list of disease-associated genes from public sources than disease-modifying drug candidates.

In an ideal situation, the gene or protein list should be taken directly from expert-curated data sources. However, for complex diseases, many disease genes, especially those associated with elevated disease risks, have not yet all been identified; moreover, the expression levels of many genes and proteins are still being investigated experimentally for potential values as “disease biomarkers” [20]. At best, researchers are often able to obtain an incomplete “initial seed list” of disease-related seed genes or proteins from heterogeneous sources. In other situations, researchers may rely entirely on known databases such as OMIM (Online Mendelian Inheritance in Man) [21] for generating an initial disease gene list. In building the AD connectivity map, we assumed users' prior incomplete knowledge on AD is derived entirely from OMIM (this assumption can be relaxed if users add supplemental genes/proteins to the seed list) and retrieved 49 AD seed proteins (corresponding to 49 genes) from OMIM. We expanded the AD seed proteins using quality-ranked protein interaction data in the OPHID (Online Predicted Human Interaction Database) [22] and a nearest-neighbor protein interaction expansion method, as described in Methods section. In the expanded AD protein interaction network, there were 560 proteins and 771 protein interactions, with confidence scores ranging from 0.30 to 1. All the 560 proteins (see Dataset S1) were ranked based on a scoring model initially described in [23] (also seen in Methods section). The scoring model assigned each of the 560 proteins an AD protein relevance score based on the protein ranking score *r_p_*. The robustness of the top protein ranks based on these scores against high levels (up to 30%) of random noise was confirmed separately for work performed subsequent to [20] (unpublished results).

In Table 1, we showed the top 30 ranked AD proteins, sorted by a descending order of the protein ranking scores derived from an AD-related protein interaction network. Among the top 30 ranked proteins, 26 were found in the initial OMIM AD seed protein list with the exception of four proteins: *APBB1_HUMAN*, *TAU_HUMAN*, *CTNB1_HUMAN* and *DAB1_HUMAN*. Two of these four proteins, *APBB1_HUMAN* and *TAU_HUMAN*, were actually present in the initial seed gene list but absent from the seed protein list after automatic gene-to-protein name conversions. This fact confirmed our findings that molecular network based gene ranking methods such as our method, CHI, ProteinRank, or CGI [23]–[26] could help recover certain biases in the initial seed list. *CTNB1_HUMAN* is a known AD protein that specifically regulates *PSEN1*, in which gene mutations can cause elevated accumulation of beta-Amyloid (*A4_HUMAN*) and lead to early-onset familial Alzheimer's Disease [27]. *DAB1_HUMAN* can be associated with the *A4_HUMAN* protein's cytoplasmic domain and causing it to over-express in hippocampal neurons—a strong indication of its key roles in AD [28].

**Table 1:** Top 30 ranked proteins from AD-related protein interaction network.

UNIPROT_ID	Description (Protein/Gene Name)	Network Degree	Rank*_NET_* Score	Occurrence	Rank*_LIT_*	Seed/Expanded
**A4_HUMAN**	Amyloid beta A4 protein precursor (APP)	48	37.031	13735	1	S
**LRP1_HUMAN**	Prolow-density lipoprotein receptor-related protein 1 precursor (LRP1)	43	34.992	716	29	S
**PSN1_HUMAN**	Presenilin-1 (PSEN1)	34	27.684	6240	3	S
**PIN1_HUMAN**	Peptidyl-prolyl cis-trans isomerase NIMA-interacting 1 (PIN1)	49	16.949	7	1638	S
**FHL2_HUMAN**	Four and a half LIM domains protein 2 (FHL2)	23	15.745	12	1244	S
**PSN2_HUMAN**	Presenilin-2 (PSEN2 )	16	14.156	1695	10	S
**S100B_HUMAN**	Protein S100-B (S100B)	14	12.165	166	184	S
**CLUS_HUMAN**	Clusterin precursor (CLU)	13	10.857	99	304	S
**NP1L1_HUMAN**	Nucleosome assembly protein 1-like 1 (NAP1L1)	84	10.185	3	2230	S
**NOG1_HUMAN**	Nucleolar GTP-binding protein 1 (GTPBP4)	101	9.601	0	–	S
**NCOA6_HUMAN**	Nuclear receptor coactivator 6 (NCOA6)	13	9.478	4	2056	S
**CDK5_HUMAN**	Cell division protein kinase 5 (CDK5)	33	8.573	633	43	S
**FLNB_HUMAN**	Filamin-B (FLNB)	10	8.464	47	551	S
**CATB_HUMAN**	Cathepsin B precursor (CTSB)	18	8.310	455	66	S
**APBA1_HUMAN**	Amyloid beta A4 precursor protein-binding family A member 1 (APBA1)	9	8.123	84	356	S
**CTND2_HUMAN**	Catenin delta-2 (CTNND2)	9	7.971	61	464	S
**ARLY_HUMAN**	Argininosuccinate lyase (ASL)	79	7.364	4	2058	S
**NCKP1_HUMAN**	Nck-associated protein 1 (NCKAP1)	6	4.860	21	913	S
**C1TC_HUMAN**	C-1-tetrahydrofolate synthase, cytoplasmic (MTHFD1)	47	4.743	0	–	S
**ODO2_HUMAN**	Dihydrolipoyllysine-residue succinyltransferase component of 2-oxoglutarate dehydrogenase complex, mitochondrial precursor (DLST)	42	4.436	508	51	S
**PRIO_HUMAN**	Major prion protein precursor (PRNP)	7	4.400	669	40	S
**RAGE_HUMAN**	Advanced glycosylation end product-specific receptor precursor (AGER)	5	4.214	258	111	S
**CTNB1_HUMAN**	Catenin beta-1 (CTNNB1)	5	4.214	328	84	E
**MK10_HUMAN**	Mitogen-activated protein kinase 10 (MAPK10)	12	4.142	24	848	S
**SNCAP_HUMAN**	Synphilin-1 (SNCAIP)	4	3.404	34	690	S
**NEU2_HUMAN**	Vasopressin-neurophysin 2-copeptin precursor (AVP)	4	3.240	160	191	S
**DAB1_HUMAN**	Disabled homolog 1 (DAB1)	4	3.240	24	845	E
**APBB1_HUMAN**	Amyloid beta A4 precursor protein-binding family B member 1 (APBB1)	4	2.641	250	115	E*
**TAU_HUMAN**	Microtubule-associated protein tau (MAPT)	3	2.595	1707	9	E*
**APLP2_HUMAN**	Amyloid-like protein 2 precursor (APLP2)	3	2.595	1473	14	S

These proteins are shown with “UniProt Entry Name” as identifiers in the **UNIPROT_ID** column. The **Node Degree** column refers to the number of proteins directly interacting with the protein of interest found in the OPHID database. The protein ranking score **Rank** ***_NET_*** **Score** is based on both network topology and quality score of each protein interaction involved, and is therefore not necessarily proportional to the protein's node degree. The proteins in this table are sorted by descending order of this score. The **Occurrence** column shows the document frequency for the protein observed from the 50,662 retrieved AD PubMed abstracts which contain the term “Alzheimer”. In the **Rank** ***_LIT_*** column, the rank of 3,130 identified proteins from the retrieved AD PubMed abstracts is determined by descending order of document frequency for each protein. The **Seed/Expanded** column indicates that a protein is in the initial seed protein set (**S**) or the network-expanded protein set (**E**), where the AD relevance of the expended ones are judged on “disease-gene” association in OMIM (“*” refers to an AD-related protein).

In Table 1, we can also determine that the disease interaction sub-network-based protein ranking result was not strongly correlated with the usage of these gene terms in the literature. The overlap between two top 500 protein lists selected from the constructed AD network and the conventional disease-specific text mining results respectively was merely 80. While *A4_HUMAN*, *PSN1_HUMAN* and *PSN2_HUMAN* were all well cited in literature and highly ranked in AD-related protein interaction network, *PIN1_HUMAN*, on the other hand, ranked fourth in the AD protein interaction sub-network yet 1,638th in the AD literature. This inconsistency suggests that there may be special opportunities in catapulting current studies of certain proteins into future prominent status that the proteins deserve. Further literature study confirms that the WW domain of *PIN1_HUMAN* binds to phosphorylated protein *TAU_HUMAN*, which is hyper-phosphorylated in AD [29]. Much more detailed semantic analysis of the PubMed abstracts would have been required to derive a comparable high-quality AD protein list without mining disease-relevant proteins in molecular interaction networks context. The high degree of disease relevance of final ranked proteins laid down a solid foundation for building a high-quality connectivity map later.

### Statistically Enriched AD-Related Drug Terms

To build the second dimension for an AD molecular connectivity map, we first retrieved PubMed abstracts of AD relevance, using the list of AD-related genes/proteins derived earlier as queries, and to parse out drug terms in the retrieved articles later. Here, we particularly withhold the urge of expediently retrieving PubMed abstracts using a conventional query term such as “Alzheimer”. Instead, we built a PubMed query with 560 AD-relevant proteins and their synonyms, and retrieved 222,609 related abstracts, without the explicit context of “Alzheimer”. The primary reason for this strategy is to improve recall of AD relevant articles. One can imagine that not all of the research studies involving 560 proteins in PubMed may be performed in the AD disease context—or in any disease context at all. For example, a biochemical study of a drug compound's effect on gene expressions would not involve any mention of AD, particularly not so in PubMed abstracts. Retrieving abstracts in any contexts based on these AD-related proteins to build an initial corpus was demonstrated to be a preferred method in improving recall of information retrieval. The recall performance was at approximately 81% (refer to Text S1 for details).

While we could build a database of all current experimental drugs and approved drugs for AD, this database would be of marginal interest to researchers focusing on novel drug discovery. Therefore, we concentrated on first identifying drug terms that are significantly “enriched” in the AD-related literature collection, as compared with the overall PubMed. In the 16,120,074 PubMed abstracts, there are 6,543 “drug chemicals” organized in a hierarchical structure, according to provided MeSH term annotations. In the 222,609 AD related PubMed abstracts retrieved from 560 AD-related proteins, 2,019 drugs remained (see Dataset S2 for a list), 1,279 of which were determined to be “enriched”—the outcome of passing a statistical term enrichment test below the pre-set filter of false discovery rate (FDR) (refer to Methods for details). Again, the associations of these significant drug terms to the “Alzheimer” disease context were made without the explicit term co-occurrence requirement for “Alzheimer” as a query term, or for particular AD genes or proteins in the same abstract. These 1,279 drugs, therefore, may constitute new knowledge worth investigation and incorporation into the AD connectivity map later.

### Assessment of Novel AD Drug Identified

**Figure 3:**
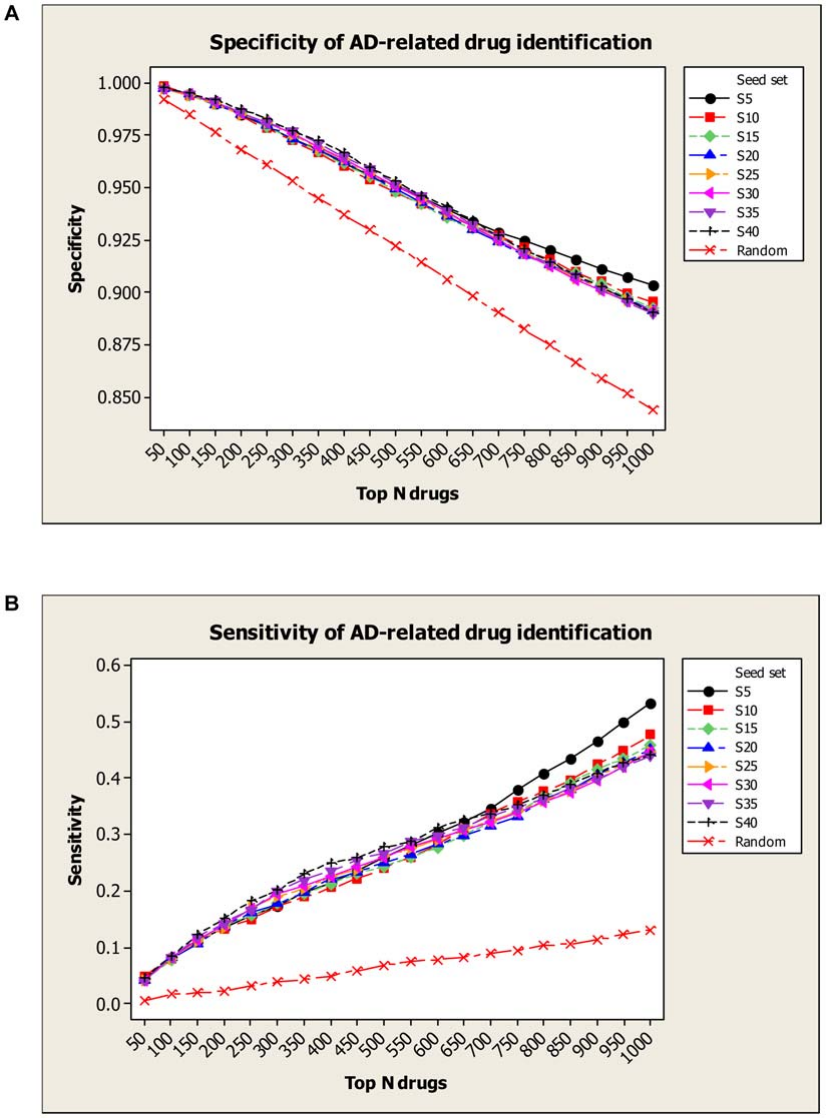
The effect of different disease-related protein seeding situation on the specificity and sensitivity of AD drug identification. In the *text retrieval and information extraction* component, the AD-related drugs are identified from the retrieved PubMed abstracts relevant to a list of AD proteins. We have an initial set of 49 AD seed proteins. To evaluate the effect of different seeding situations on AD drug identification, we sub-sampled the initial AD seed set into 8 data sets of varying sizes i.e., S5, S10, S15, S20, S25, S30, S35, S40 (the number indicating size) and also generated a random seed set with 50 proteins.. Given different seed sets, Panel (A) shows the specificity performances of AD-related drug identification at top N drugs determined by FDR (false discovery rate), and Panel (B) shows the sensitivity performances.

To estimate how the first stage (the *network construction* component) affected the second stage (the *text retrieval and information extraction* component), we evaluated the performance of AD-related drug identification by changing the input of AD seed proteins. Given different sets of the initial seed proteins, we calculated sensitivity and specificity at top N drugs determined by FDR (refer to Methods section). In summary, we sub-sampled 49 AD seed proteins into 8 data sets of varying sizes *i.e.*, S5, S10, S15, S20, S25, S30, S35, S40 (the number indicating size) and also generated a random seed set with 50 proteins. As shown in Figure 3, the overall specificity and sensitivity are robustly maintained while the seed set changed from S5 to S40 (overall specificity variance<0.000021 and sensitivity variance<0.00098). The random seed performance was distinctly lower than any seeding strategies experimented. This shows that potential bias in selecting seed proteins did not significantly affect drug identifications. We attribute this success to the molecular interaction network approach used.

**Figure 4:**
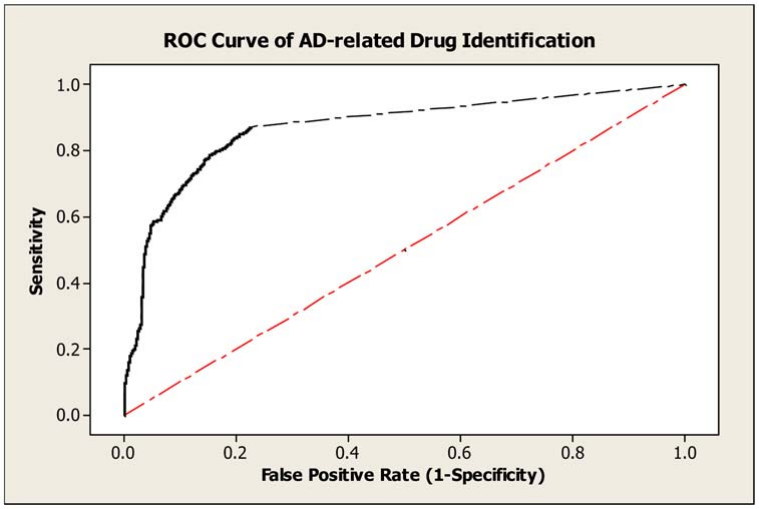
Specificity and sensitivity tradeoffs for AD-related drug identification. The ROC (receiver operating characteristic) curve shows the sensitivity vs. false positive rate (1-specificity) for AD-related drug identification, when FDR (false discovery rate) varies at different threshold levels. Evaluation results are built by querying against PubMed abstracts and Enrez gene function description in search of evidence that may contain any of the drug terms and the term “Alzheimer's Disease” with all their term variants. The sensitivity and specificity are defined in Methods section.

We evaluated the overall performance of our drug enrichment method using a Receiver Operating Characteristic (ROC) curve (Figure 4). We used the 49 AD seed proteins as the initial input, and calculated sensitivity and specificity tradeoffs by varying different FDR thresholds over the entire 2,019 identified drugs against a “gold standard” of AD drugs (see Methods for details). The best sensitivity was seen at 87.2% based on the result. When we set FDR threshold at 0.05, the system achieved an overall sensitivity of 76.0% and a specificity of 88.8%. This confirmed our literature mining approach to be satisfactory for practical use.

Then, we investigated the 1,279 AD-related drugs in detail. At FDR<0.05, 50.1% of 1,279 drugs can be found also in the 843 “positive” AD drugs of the “gold standard”; therefore, the positive predictive value (PPV) of the drug enrichment test was 50.1%. Compared to the general prevalence (843/6,543 = 12.8%) of AD drugs, the AD drug enrichment method was able to enrich AD-related drugs almost fourfold (50.1%/12.8% = 3.91). We believe the 4× enrichment and 50.1% PPV were conservative estimates, because the construction of “gold standard” AD drugs did not take into account potentially novel AD drugs. In Table 2, we listed 25 representative drugs from the total collection of 2,019 drugs found in 222,609 AD-related PubMed abstracts. Among the tabulated 22 drugs with FDR<0.05, 17 were confirmed to be AD-related determined by us reading literature. Among the 17 drugs, *Rivastigmine*, *Tacrine*, *Donepezil*, *Vitamin E*, *Memantine*, and *Galantamine* are approved drugs for AD treatment in DrugBank [30]. Some enriched drugs are predominantly known for other disease treatments, such as *Fenoldopam* is a vasodilator that may reduce blood pressure in hypertension and decrease both regional and global cerebral blood flow; *Droxidopa* is a precursor of noradrenaline, which is used in the treatment of Parkinson and Familial Amyloidotic Polyneuropathy—both related to AD. About the repurposed drug investigation, it was separately discussed in the subsequent subsection. The last two examples showed that novel AD drug candidates may be derived by our method by automated implicit knowledge transfer from disorders with symptoms closely related to AD. We also observed that drugs occurring more frequently in the retrieved PubMed abstracts yet with high FDR values are indeed not specific to AD, for example, *Glycerol* (Cryoprotective Agents; DF_AD = 185) and *Tetracycline* (Protein Synthesis Inhibitors; Anti-Bacterial Agents; DF_AD = 151). Although both are highly mentioned in the AD-related articles as common chemicals used to process biological samples, they are filtered out of our enriched drug list for further AD connectivity maps construction.

**Table 2:** A representative sample of enriched AD drugs.

Drug	FDR	DF_AD	Pharmacological Action	Found In AD-GS	Found In DrugBank
**Rivastigmine**	<1.00E-16	571	Cholinesterase Inhibitors; Neuroprotective Agents	Yes	Yes
**Bromocriptine**	<1.00E-16	139	Antiparkinson Agents; Dopamine Agonists; Hormone Antagonists	Yes	
**Fenoldopam**	<1.00E-16	11	Antihypertensive Agents; Vasodilator Agents; Dopamine Agonists		
**Diazepam**	<1.00E-16	149	Adjuvants, Anesthesia; Anti-Anxiety Agents; GABA Modulators; Anesthetics, Intravenous; Muscle Relaxants, Central; Anticonvulsants; Antiemetics; Hypnotics and Sedatives	Yes	
**Tacrine**	2.40E-15	31	Cholinesterase Inhibitors; Parasympathomimetics; Nootropic Agents	Yes	Yes
**Donepezil**	5.24E-15	16	Cholinesterase Inhibitors; Nootropic Agents	Yes	Yes
**Dopamine**	3.87E-12	966	Dopamine Agents; Cardiotonic Agents	Yes	
**Calcimycin**	3.87E-12	325	Anti-Bacterial Agents; Ionophores	Yes	
**Vitamin E**	3.87E-12	198	Antioxidants; Vitamins	Yes	Yes
**1-Methyl-3-Isobutylxanthine**	3.87E-12	192	Phosphodiesterase Inhibitors	Yes	
**Theophylline**	3.87E-12	144	Bronchodilator Agents; Phosphodiesterase Inhibitors; Vasodilator Agents	Yes	
**Ethidium**	3.87E-12	58	Enzyme Inhibitors; Fluorescent Dyes; Trypanocidal Agents; Indicators and Reagents; Nicotinic Antagonists	Yes	
**Flunitrazepam**	3.87E-12	37	GABA Modulators;Anti-Anxiety Agents	Yes	
**Apomorphine**	3.87E-12	117	Antiparkinson Agents; Dopamine Agonists	Yes	
**Diltiazem**	3.87E-12	63	Antihypertensive Agents; Vasodilator Agents; Cardiovascular Agents; Calcium Channel Blockers		
**Prazosin**	3.87E-12	129	Adrenergic alpha-Antagonists; Antihypertensive Agents	Yes	
**Quinidine**	1.68E-08	27	Anti-Arrhythmia Agents; Adrenergic alpha-Antagonists; Enzyme Inhibitors; Muscarinic Antagonists; Antimalarials		
**Clonazepam**	9.04E-07	21	Anticonvulsants; GABA Modulators	Yes	
**Memantine**	2.24E-06	8	Dopamine Agents; Excitatory Amino Acid Antagonists; Antiparkinson Agents	Yes	Yes
**Droxidopa**	6.04E-05	5	Antiparkinson Agents		
**Dextrorphan**	7.01E-05	6	Excitatory Amino Acid Antagonists; Neuroprotective Agents		
**Galantamine**	0.039622	6	Cholinesterase Inhibitors; Nootropic Agents; Parasympathomimetics	Yes	Yes
**Glycerol**	0.085	186	Cryoprotective Agents	Yes	
**Tetracycline**	0.14	151	Protein Synthesis Inhibitors; Anti-Bacterial Agents	Yes	
**Amikacin**	0.86	28	Anti-Bacterial Agents		

This table includes all the drugs mentioned in this manuscript (see Dataset S2 for the completed version). The **FDR** (false discover rate) value for each drug is shown in this table. The **DF_AD** column provides each drug's document frequency in the retrieved PubMed abstract corpus. The **Pharmacological Action** column lists MeSH annotated Pharmacological information for each drug. The **Found In AD-GS** column indicates whether a drug retrieved is also found in the Gold Standard (GS) data set for AD. The **Found in DrugBank** column indicates whether a drug retrieved is also found in the DrugBank database. Note that drugs listed here are not yet applied with any minimal FDR threshold filter.

**Figure 5:**
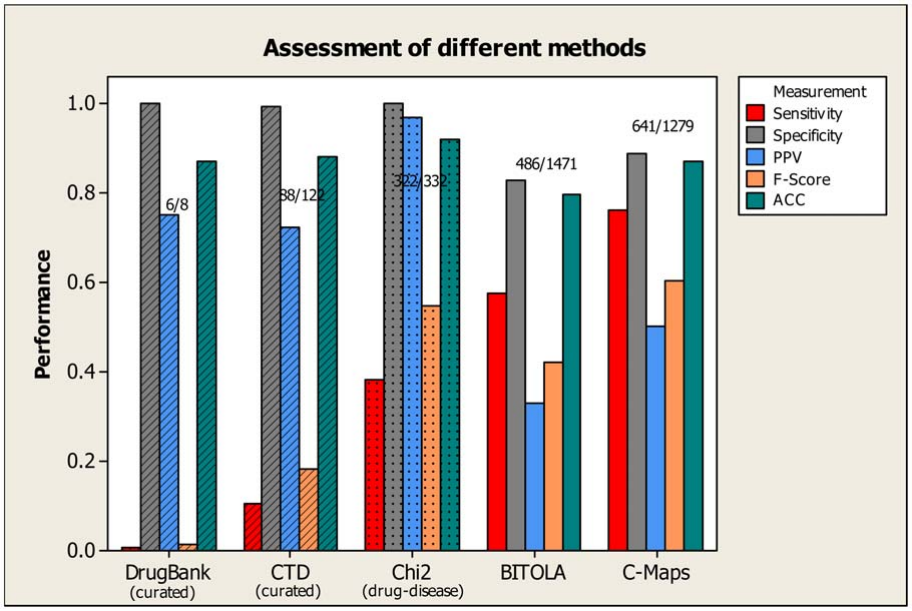
Performance assessment of comparable systems on the task of identifying AD-related drugs. Two curated data sources (DrugBank and CTD) and two computational methods (Chi2 and BITOLA) were selected to compare against the performance of our approach on AD drug identifications. DrugBank and CTD manually curated database content about disease-modifying gene/proteins and drugs. Chi2 is a baseline system using commonly Chi-square statistical method to identify significant co-occurring drug-disease relationships cited in PubMed abstracts. BITOLA (Biomedical Discovery Support System) is a computational system based on natural language processing that can extract drug-protein relation in a disease context. The histogram shows sensitivity, specificity, PPV (positive predictive value), F-score, and ACC (accuracy) of each group. These performance measurements are defined in the Methods section.

Lastly, we compared the performance of several related systems, which also aims to provide information mapping diseases, drugs, and genes/proteins, and showed this result in Figure 5. The four systems that we compared includes: DrugBank [30], CTD (the Comparative Toxicogenomics Database) [31], a baseline system using the common Chi-square (χ^2^) method [32], and BITOLA (Biomedical Discovery Support System) [17]. DrugBank is a manually annotated resource that combines detailed drug data with drug target information such as protein sequence, structure and pathway. It includes FDA approved small molecule, biotech (peptide or protein), nutraceutical, withdrawn, illicit, and experimental drugs. Only eight AD drugs were reported in the DrugBank database. Since it only contains known drugs currently approved or experimented for a disease, its specificity (∼100%) and PPV (75%) were understandably better than ours. However, since Drugbank does not contain candidate drug information, its sensitivity (0.7%) and F-Score (1.4%) were extremely low. Similar to Drugbank, CTD is a manually curated data resource that includes cross-species chemical-gene/protein interactions, chemical-to-disease relationships, and gene-to-disease relationships. Since it also only contains known therapeutic drug information, its high specificity (99%) and PPV (72%) were also accompanied by low sensitivity (10.4%) and F-Score (18.2%). Commonly designed baseline text mining systems using standard Chi-square statistic can also offer a measure of significant drug-disease association explicitly stated in PubMed abstracts. Applying a threshold (*p-value*<0.05) in Chi-square test, we could derive 332 AD candidate drugs. Since these drugs are already co-cited with AD, its specificity (∼100%) and PPV (∼100%) were understandably high (and included as part of the gold standard), however, many potentially novel drugs would also be missed as a result of meeting the disease-drug co-currence requirement, causing such systems to suffer badly in sensitivity as compared to our method (38% Chi-square vs. 76% ours). BITOLA is an advanced system that can automatically extract semantic relations between biomedical concepts, built on interactive nature language processing techniques. To compare BITOLA against our approach, we queried BITOLA server with AD as a disease to retrieve a list of AD genes/proteins first and iteratively retrieved AD drugs later. BITOLA retrieved 1,472 AD candidate drugs. Its specificity (82.7%), PPV (33.0%) and accuracy (79.5%) were lower than all three systems described earlier but its sensitivity (57.7%) and F-Score (42.0%) were better. In comparison, our approach performed better than BITOLA in all of these performance measures, with a balanced F-Score (60.4%) outperforming all four.

### Creation and Assessment of AD Connectivity Map

With sufficient number of proteins and drugs enriched on disease relevance from molecular interaction networks and biomedical literature, we were ready to build a connectivity map with balanced quality and coverage. The AD connectivity map matrix was built with proteins as rows, drugs as columns, and a protein-drug connectivity score based on co-citation adjusted log-odds values (see Methods for details). Two dimensional hierarchical clustering was applied to identify groups of proteins/drugs sharing similar profiles. In the AD connectivity map, proteins were clustered between similar drug profiles and drugs were also clustered between similar protein profiles.

To assess the biological significance of drug-protein connectivity scores, we compared high-scoring protein-drug pairs in the AD connectivity map with all the known AD drug-target relations in DrugBank. Since only 6 out of 8 AD drugs reported in DrugBank were involved in our study (see Figure 5), we collected all the 6 drugs' target proteins from the DrugBank database for the comparison. Table 3 showed all high-scoring protein-drug connectivity pairs with connectivity score >1.We used the concept of “target distance” to measure between a drug-protein connectivity profile created and the actual drug-target knowledge. More precisely, we defined target distance as the shortest distance in the disease-specific protein interaction sub-network between a drug's target protein in DrugBank and the drug's connected protein in the molecular connectivity map. A target distance of 0 refers to a protein in the molecular connectivity map also to be the drug's target protein. *Tacrine* and *Galantamine* targeted their connected protein (*ACES_HUMAN*) directly, which covered four proteins listed (*ACES_HUMAN*, *CATB_HUMAN*, *A4_HUMAN*, *EP300_HUMAN*). *Vitamin E* seemed to contain several long-range connections to AD proteins, with a target distance of 2. *Memantine* seemed to be the only example with the farthest known path of protein interactions to its target (target distance = 3). All the four highly associated known AD drugs are within a target distance of 3.

**Table 3:** Cross-validation of protein-drug relationships identified in the AD connectivity map with target-drug relationships of AD drugs in Drugbank.

UNIPROT_ID	Drug	Connectivity Score	Target Distance*
ACES_HUMAN	Tacrine	4.77	0
ACES_HUMAN	Galantamine	4.22	0
MK_HUMAN	Memantine	4.12	3 (*NMDE1-SRC-ETS1-MK*)
CATB_HUMAN	Tacrine	3.46	2 (*ACES-A4- CATB*)
A4_HUMAN	Tacrine	3.40	1 (*ACES-A4*)
EP300_HUMAN	Tacrine	3.17	2 (*ACES-A4-EP300*)
TTHY_HUMAN	Vitamin E	1.31	2 (*KPCA-NMDE1-TTHY*)
MK08_HUMAN	Vitamin E	1.09	2 (*KPCA- NMDE1-MK08*)

*: A shortest protein interaction path in the AD protein interaction subnetwork consisting of all AD-related proteins is indicated.

### Exploration of AD Connectivity Map

**Figure 6:**
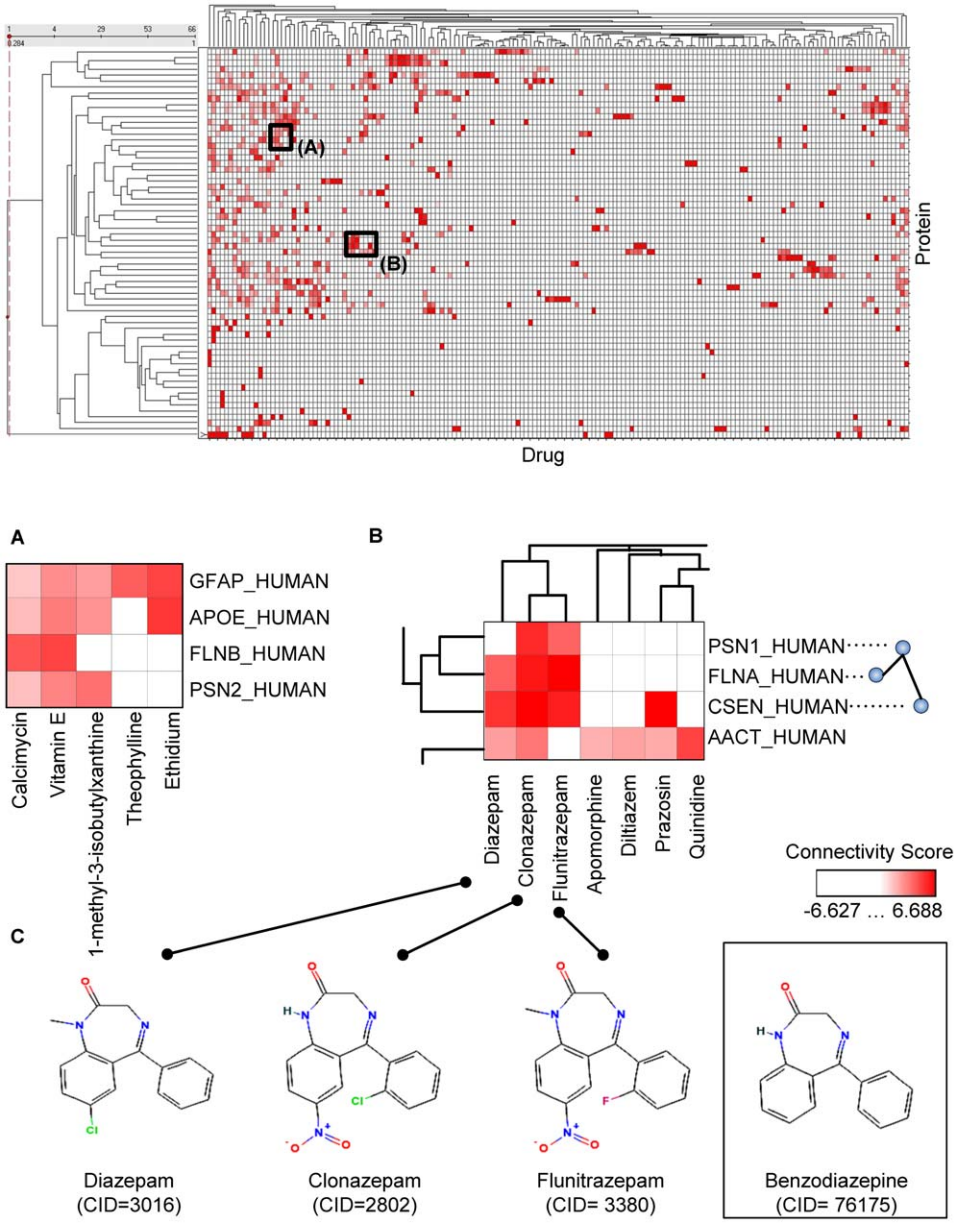
An AD connectivity map linking AD-related proteins to significant drugs. After ranking proteins involved in the AD related protein interaction network and selecting enriched drugs in AD network related corpus, 66 AD highly-relevant proteins and 166 significant AD candidate drugs are identified to construct an AD connectivity map. Hierarchical clustering of drugs and proteins are performed before results are shown as the final heatmap format, in which the *x*-dimension represents drugs and the *y*-dimension represents proteins. The color intensity for each cell is drawn in proportion to the connectivity score as shown in the heatmap legenda. Panels (A) and (B) show zoomed-in views of boxed regions A and B on the original map. Panel (C) shows the chemical structures of three drugs (*Diazepam*, *Clonazepam*, and *Flunitrazepam*) from a cluster of drugs found in Panel (B), with their common structure (*Benzodiazepine*) shown in a box. CID refers to entity identifier in PubChem (http://pubchem.ncbi.nlm.nih.gov/).

In the AD connectivity map (see Figure 6 and its source data in Dataset S3), the connections between 166 drugs and 66 proteins could be reviewed globally or queried for each pair of drug and protein/gene. The AD connectivity map contained a wealth of information worth investigating by biomedical researchers. For example, in Figure 6A, many high-lighted cells were supported by existing knowledge, including 1) “*GFAP_HUMAN* vs. *Calcimycin*”, where it was reported that in the central nervous system related diseases, the treatment with the calcium ionophore (*calcimycin*) resulted in a rapid increase in immunoreactivity to glial fibrillary acidic protein (*GFAP_HUMAN*) [33]; 2) “*GFAP_HUMAN* vs. *Vitamin E*”, where the increased expression of *GFAP* during astrocyte differentiation could be mediated by *vitamin E* administration [34]; 3) “*APOE_HUMAN* vs. *1-Methyl-3-isobutylxanthine* (*IBMX*)”, where *IBMX* treatment increased *cAMP* levels and diminished *cGMP* levels in the absence of *apoE* complex [35].

In the connectivity map, proteins that interact with each other seemed to cluster well with each other based on added protein-drug profile similarities. For example, in Figure 6B, *PSN1_HUMAN*, *FLNA_HUMAN* and *CSEN_HUMAN* shared highly similar protein-drug connectivity profiles among themselves. According to HPRD [36], *PSN1_HUMAN* directly interacts with both *CSEN_HUMAN* and *FLNA_HUMAN*. This may be a factor in explaining why drugs intervening *PSN1_HUMAN* may affect *CSEN_HUMAN* and *FLNA_HUMAN* as well. Also, drugs *Diazepam*, *Clonazepam*, *Flunitrazepam*, *Apomorphine*, *Diltiazem*, *Prazosin*, and *Quinidine* were clustered closely. When their chemical structures were examined, we found them to share common two-ring structures. *Diazepam*, *Clonazepam* and *Flunitrazepam* were further found to contain *benzodiazepine* as a common structure (see Figure 6C). Another interesting observation on this group of drugs was their shared similar pharmacological actions: *Diazepam*, *Clonazepam* and *Flunitrazepam* are for the symptomatic treatment of anxiety disorders, while *Diltiazem* and *Prazosin* are for the treatment of vascular hypertension. These findings suggest that a decent degree of accuracy may have been achieved to enable one to drill down to underlying mechanisms, biologically or chemically, between connected molecules.

### Investigation of Repurposed Candidate Drugs from the AD Connectivity Map

Disease-specific molecular connectivity maps can provide novel insights for re-purposing experimental drugs, successful or failed, from the original intended therapeutic area to a new disease application context. In the above example as shown in Figure 6B, *Diltiazem*, *Prazosin* and *Quinidine* were clustered together due to their similar drug-protein connectivity profiles. The three drugs are previously known to treat vascular diseases. Among them, *Diltiazem* is an antihypertensive agent with vasodilating actions due to its antagonism of the actions of the calcium ion in membrane function; *Prazosin* is an alpha-adrenergic blocking agent used in the treatment of heart failure and hypertension; *Quinidine* is an anti-arrhythmia agent with actions on sodium channels on the neuronal cell membrane. Recent population-based epidemiological studies suggested that vascular risk factors, such as vascular disease gene *ApoE* [37],[38], hypertension [39], atherosclerosis [40], and heart failure [41], may impair cognitive functions and are related to the development of AD. Both randomized and non-randomized clinical trials indicated that lowing blood pressure could play an important role in preventing AD. Further trials also demonstrated that antihypertensive agents decrease the incidence of dementia in stroke patients ([42],[43]) and in elderly patients with isolated systolic hypertension [44]. Additionally, a latest research study showed that *Valsartan*, an anti-hypertensive chemical, can reduce AD-like symptoms in mice [45]. Not too surprisingly, when we look into clinical trial databases, we found that *Prazosin* is currently under a double-blind and placebo-controlled clinical study on the treatment of agitation and aggression in persons with AD (ClinicalTrials.gov Identifier = NCT00161473) [46], while *Diltiazem* and *Quinidine* have not been experimented for any AD related treatment to our best knowledge. Could *Diltiazem* and *Quinidine* become worthwhile candidates for future AD drug re-purposing investigation? With newly-derived molecular connectivity maps guiding drug developers to hypothesize on therapeutic values of these two candidate drugs, we believe the answer is “yes”. We believe there are many drug candidates in our results beyond what presented here that are also worthwhile experimenting.

## Discussion

In this study, we developed a novel computational framework to build drug-protein molecular connectivity maps. Our approach integrated protein interaction network mining and text mining, without the need for gene expression data sets derived from drug perturbation experiments on disease samples. We showed that this approach was effective in constructing a statistically significant AD molecular connectivity map that links AD-related proteins to AD-related drugs with enriched PubMed literature references. This work pointed out a new direction for biomedical researchers to integrate functional contexts of proteins from molecular interactions networks with published literature studies on drugs. The resulting AD connectivity map consisted of comprehensive collections of AD drug-protein connectivity profiles, which may help guide hypothesis generations of AD drug development.

While we focused on a case study of Alzheimer's disease in this paper, our approach has shown similarly robust performance for many other disease applications. In Table 4, we showed a comparison of performance measures of our approach for six representative cancers. The result confirmed that molecular connectivity maps can be developed for a broad range of disease applications at a comparable performance to AD. The performance varied among different diseases, primarily due to varying degree of prior knowledge and varying amount of biomedical studies focused on different diseases. For example, breast cancer is a well-studied disease compared with pancreatic cancer. More biomedical reports and more treatment progress in breast cancer study are available than those in pancreatic cancer. This is a primary reason that the F-Score performance was higher in breast cancer (56.3%) than in pancreatic cancer (46.2%).

**Table 4:** Performance assessment of molecular connectivity maps for several representative cancers.

	Breast	Pancreas	Leukemia	Lung	Ovary	Prostate
**Sensitivity**	71.3%	68.5%	63.5%	57.8%	70.0%	61.4%
**Specificity**	88.0%	89.4%	88.5%	91.4%	87.8%	91.6%
**PPV**	46.5%	34.8%	52.1%	52.1%	31.4%	40.1%
**F-Score**	56.3%	46.2%	57.2%	54.8%	43.3%	48.5%
**ACC**	85.8%	87.8%	84.4%	86.6%	86.5%	89.1%

The table shows a comparison of performance measures—sensitivity, specificity, PPV (positive predictive value), F-score, and ACC (accuracy)—for six major cancer drug identification tasks. The evaluation procedure and the development of “gold standard” for each cancer study follows the same method developed for AD. The complete set of all the drugs identified for the 6 cancers is shown in Dataset S4, which contains information on False Discover Rate (FDR), Document Frequency (DF) in the retrieved PubMed abstract corpus, Pharmacological Action, and validation flags (Found In GS and Found In DrugBank) for each drug.

Several factors contributed to the effectiveness of our new framework to develop computational molecular connectivity maps. First, we considered all available biomedical abstracts in PubMed as a primary source of data, therefore potentially incorporating all published knowledge of genes, proteins, drugs, and diseases. Second, we applied a molecular network mining method to prioritize disease-specific genes/proteins, therefore making use of molecular functional link information embedded in high-throughput protein interactomes [47]. Third, we used disease-specific genes/proteins to extract indirect relationships between diseases and drugs, therefore providing opportunities for discovering new therapeutic applications of existing drugs. Fourth, we applied advanced statistical techniques to rank disease-related protein in a disease specific protein interaction subnetwork, rank enriched drug terms from retrieved PubMed abstract collection, and score protein-drug associations based on significant term co-occurrence–which collectively increased data processing efficiency and reduced error rates (refer to Text S2 for details).

We have implemented a web server (http://bio.informatics.iupui.edu/cmaps), which allows users to query and explore molecular connectivity maps developed using methods described here. Users of the web server can input a query disease name, for example, *Alzheimer*, and the web server can suggest further standard MeSH disease ontology terms such as “*Alzheimer Disease*” or “*Acute Confusional Senile Dementia*” before showing connectivity map data for a specific disease chosen by the user. The connectivity map data is shown in a html table, populated with statistically significant protein-drug association pairs. Users can navigate through the data's hyperlink to web pages that contain detailed annotation information on the protein (e.g., “*A4_Human*”), the drug entity (*e.g.*, “*Tacrine*”), or literature abstract where the protein and drug terms are highlighted in the same abstract context. Further description of the web server has been planned in a separate manuscript beyond the scope of this work.

Ongoing research to develop molecular connectivity maps of higher coverage and confidence, particularly when applied to other therapeutic disease areas, may present new opportunities for biomedical researchers to perform integrative bioinformatics and cheminformatics for future drug discoveries. Further improvement of molecular connectivity map data accuracy may be achieved by integrating genomics, functional genomics, and proteomics experimental data to build better disease seed genes/proteins, incorporating diversified types of molecular interaction network data of growing coverage and quality, and collecting full articles instead of abstracts related to disease's molecular mechanism. Future researchers may explore shifting trends of different such maps that are to be built over different temporal dimensions, among literature sub-collections of journal within different readership and impacts, and under different biological experimental conditions. Results from our approach may be integrated with experimental gene expression or protein expression data, as they become available, to improve thorough classification of the type of associative relationships hidden from the drug-protein connectivity maps. Molecular connectivity maps that connect protein and metabolites could also be developed. Protein-metabolite molecular connectivity maps in model organisms may further facilitate comparative genomics analysis. Chemical biologists may further investigate the relationships between common chemical sub-structures and common protein structure motifs for drug compound optimizations. A software server that employs molecular connectivity mapping concepts will enable users to gain comprehensive knowledge of drug-protein connectivity profiles, compare chemical compounds based on their functional connectivity profile similarities, and drill down to specific PubMed articles for details. Our study presents many further research opportunities in post-genome drug development.

## Methods

### Construct Disease-Related Protein Interaction Network

In the network construction component, we adopted a method originally developed by Chen *et al.* [23] to construct disease-related protein interaction network and a ranked list of disease-relevant proteins. The disease-related seed genes/proteins may be provided by disease biology users or found in the OMIM database. We used the Online Predicted Human Interaction Database (OPHID) [22] to collect human protein interaction data and construct protein interaction sub-networks. OPHID is a protein interaction database containing 1) literature-derived interaction data from HPRD [36], BIND [48] and MINT [49]; 2) human interaction data from high-throughput experiments; and 3) transferred interactions from orthologous proteins in eukaryotic model organisms. While 23,889 of 39,923 interactions are predicted interactions, the quality of the OPHID data set can be controlled for successful disease-specific studies [23],[50],[51]. George, *et al.* examined the OPHID protein interaction data set, and used it to predict candidate disease genes. They assessed the usefulness of protein interactions from different OPHID protein interaction data categories. They built a successful case to show that the full OPHID dataset was applicable to disease-related studies [51]. Chen, *et al.* also used OPHID full data set, but by assigning them different confidence scores based on the source of the data. Using the weighted protein interaction data, they successfully constructed an AD related sub-network and validated the disease relevance of the top-ranked proteins in the sub-network [23]. Here, we adopted a similar weighted approach when using OPHID data sets, and a similar ranking method when calculating protein's disease relevance score, *r_p_*, as the following:
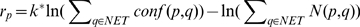
(1)


In the formula, *p* and *q* are indices for proteins in the disease-related interaction network *NET*. *k* is an empirical constant (*k* = *2* in this study). *conf(p, q)* is a confidence score assigned to each interaction *(p, q)* between protein *p* and *q*. Consistent with [23], *conf(p, q)* = 0.9 if *(p, q)*∈{curated interactions}, *conf(p, q)* = 0.5 if *(p, q)*∈{predicted interactions from mammalian organisms}, and *conf(p, q)* = 0.3 if *(p, q)*∈{predicted interactions from non-mammalian organisms}. *N(p, q)* holds the value of 1 if the protein *p* interacts with *q*. The *r_p_* score is used to rank proteins and filter out protein-drug associations that may arise due to noise in literature mining results.

### Determine and Select Enriched Drugs

We used a term frequency statistical method to take advantage of term statistical distributions from the entire PubMed abstracts, and calculated the *p*-value of each term's significance in being observed in any collection of retrieved PubMed abstracts [52]. The main reason for doing so is to control false positives among terms determined to be significantly enriched. For example, observing abnormally high usage frequency of a term from *tf-idf* could lead to the incorrect inclusion of the term as “enriched”, because the sampled document subset could be biased, and the term usage frequency could be intrinsically variable.

In this work, we first retrieved the entire PubMed abstracts using the expanded list {*p_1_*, *p_2_*,…, *p_m_*} containing all the proteins in a network as the initial query [53]. From the retrieved abstract collection *T_NET_*, the drugs {*d_1_*, *d_2_*,…, *d_n_*} were then identified automatically by our system combining both dictionary and rule directives. We calculated a *p-value* for each drug *d_j_* in *T_NET_* using methods described in [53] and later derived its false discovery rate. Let the null hypothesis H_0_ be that document frequency of drug *d_j_* in *T_NET_* come from a random distribution. The *t*-test value *Δ_j_* for drug *d_j_* can then be calculated as:
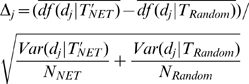
(2)


Here, 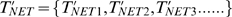 is generated by sampling the entire collection of retrieved document abstracts 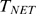 (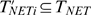, 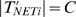 is a predefined number of documents) and 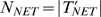 is the size of each sample. 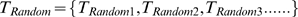 refers to a random sample generated by randomly sampling the entire PubMed abstracts and the size of the random sample is 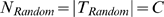 (we set *C to* 1000 to keep it consistent with non-random sample sizes). 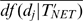 and 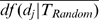 refer to average document frequencies of *d_j_* in 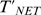 and 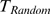. 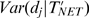 and 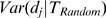 refer to document frequency variances of *d_j_* in 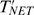 and in 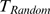. The *p*-value is computed as from two-sided tails P(|*Z*|>|Δ|), where *Z*∼N(0, 1)):
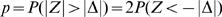
(3)


We used a standard multiple testing correction method [54] in microarray analysis to convert *p*-values from the *t*-test to calculate a drug's false discovery rate (FDR). In the end, enriched drugs {*d_1_*, *d_2_*,…, *d_g_*} were the ones that met an empirically determined threshold (term frequency >4 and FDR<0.05).

### Connect Protein and Drug for Specific Disease

We assigned a connectivity score *Θ* for each possible pair of ranked proteins {*p_1_*, *p_2_*,…, *p_k_*} from user inputs and enriched drugs {*d_1_*, *d_2_*,…, *d_g_*} , using a regularized log-odds function as following. The log-odds framework was able to qualify association strengths, in particular, facilitated the handling of words for which only sparse scientific literature existed [55].
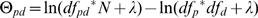
(4)


Here, *df_p_* and *df_d_* are the total number of documents in which protein *p* and drug *d* are mentioned, respectively, *df_pd_* is the total number of documents in which protein *p* and drug *d* are co-mentioned in the same document. *N* is the size of the entire PubMed abstract collection. λ is a small constant (λ = 1 here) introduced to avoid out-of-bound errors if any of *df_p_*, *df_d_*, or *df_pd_* values are 0. The resulting *Θ_pd_* is positive for when the protein-drug pair is over-represented and negative when the protein-drug is under-represented. The higher the *Θ_pd_* is, the more significant the over-representation of connection becomes. Totally, *k* x *g* connectivity scores were calculated to build connectivity map.

### Evaluation of AD-Related Drug

A “gold standard” of 843 AD-related drugs was constructed using one of the following criteria: (1) **Co-citation in the PubMed abstracts**: a drug term and all its term variants co-occur with the phrase “Alzheimer's Disease” in at least two PubMed abstracts. In other words, we assume that a drug should be related to a disease if it is co-cited with the disease term in more than one article (one may tighten or loosen this criterion in other disease applications). (2) **Co-occurrence in GeneRIF sentences**: a drug term and all its term variants co-occur with “Alzheimer's Disease” in at least one gene function annotation GeneRIF entry in the Entrez Gene database [56]). Here, we assume GeneRIF to contain higher quality information than general PubMed abstracts when it is used to describe the function of a specific gene.

The “gold standard” should not be mistaken for “true confirmed drugs with therapeutic or toxicological values”. Instead, it provides an executable, balanced, and unbiased disease-related drug list for *performance evaluation purpose* only. In the above automated method for AD “gold standard” construction, we used coverage and disease-relevance as the most important criteria, considering both peer-reviewed article abstracts and curated gene function annotations from reputable databases.

The following measurements were involved in our evaluation and comparison experiments. (1) **Sensitivity** is the percent of correctly identified AD-related drugs; (2) **Specificity** is the percent of correctly identified non AD-related drugs; (3) **PPV (Positive Predictive Value)** is the probability of correct positive prediction; (4) **F-score** is the harmonic mean of Sensitivity and PPV; (5) **Accuracy** is the proportion of correctly predicted drugs.
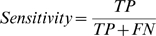
(5.1)

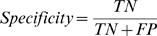
(5.2)

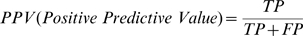
(5.3)

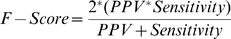
(5.4)

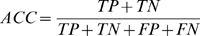
(5.5)


### Clustering of Proteins or Drugs in a Disease-Specific Molecular Connectivity Map

In the integrated analysis component, two-dimensional hierarchical clustering of the drug-protein connectivity map was performed using the weighted pair-group method and arithmetic mean method, with Tanimoto as similarity measures. The similarity between two drugs *d_a_* and *d_b_* was calculated as following:
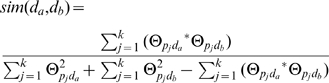
(6)where, 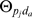 and 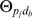 are cell values calculated by the function 4. The similarity between proteins was also calculated by the function 6.

The final clustered attributes along the drug dimension (horizontal axis) and protein dimension (vertical axis) were sorted by averaged values, decreasing from left to right and from top to bottom. The clustering was performed and visualized with the Spotfire DecisionSite Browser 8.2 software. The tool has been widely used in bioinformatics.

## Supporting Information

Dataset S1 The table contains 560 proteins with their network degrees and ranking scores in the AD-related protein interaction network.
(0.06 MB XLS)

Dataset S2 The table contains 2,019 drugs identified from 222,609 PubMed abstracts related to AD protein interaction network. It also contains the False Discover Rate (FDR), document frequency in the AD related corpus (DF_AD), Pharmacological Action, and validation flag for each drug.
(0.33 MB XLS)

Dataset S3 The file includes one source matrix data of Figure 6 (a connectivity map for Alzheimer's Disease connecting 66 proteins with 166 drugs), and one table containing protein-drug associations shown pairwise with their evidence PMIDs.
(0.32 MB XLS)

Dataset S4 The file includes the complete drug lists for breast cancer, pancreatic cancer, leukemia, lung cancer, ovarian cancer and prostate cancer. Like Dataset S2 for AD, it contains False Discover Rate (FDR), Document Frequency (DF) in the retrieved PubMed abstract corpus, Pharmacological Action, and validation flags (Found In GS and Found In DrugBank) for each drug.
(1.13 MB XLS)

Text S1 Information Retrieval Performance and Method
(0.08 MB PDF)

Text S2 A Discussion of Statistical Components Used
(0.09 MB PDF)
