# Supplementary material for: Building Disease-Specific Drug-Protein Connectivity Maps from Molecular Interaction Networks and PubMed Abstracts
Source: PLoS Comput Biol. 2009 Jul 31;5(7):e1000450. doi: 10.1371/journal.pcbi.1000450 (PMC2709445; doi:10.1371/journal.pcbi.1000450)
Supplement: Text S1 — Information Retrieval Performance and Method (0.08 MB PDF) [file pcbi.1000450.s005.pdf]

Information Retrieval Performance and Method

Enhanced Recall Performance of AD PubMed Abstract Retrieval

To build a list of AD-related drugs for a connectivity map, we first retrieved PubMed abstracts of AD relevance, using the list of AD-related genes/proteins derived earlier as queries, and to parse out drug terms in the retrieved articles later. Here, we particularly withhold the urge of expediently retrieving PubMed abstracts using a conventional query term such as "Alzheimer". Instead, we built a PubMed query with 560 AD-relevant proteins and their synonyms to retrieve abstracts, without the explicit context of "Alzheimer". The primary reason for this strategy is to improve recall of AD relevant articles. One can imagine that not all of the research studies involving 560 proteins in PubMed may be performed in the AD disease context—or in any disease context at all. For example, an analytical chemistry or biochemistry study of a drug compound's effect on gene expressions would not involve any mention of AD, particularly not so in PubMed abstracts. Therefore, retrieving as many abstracts as possible in any context (high recall) based on these AD-related proteins to build an initial corpus should be a preferred method in improving recall. Note that the 560 AD proteins were expanded with a comprehensive dictionary of gene/protein name thesaurus and a flexible query syntax that allows imprecise name entity recognitions to further improve recall.

Figure S1. **Information retrieval performance of PubMed abstracts based on protein/gene term as queries.**

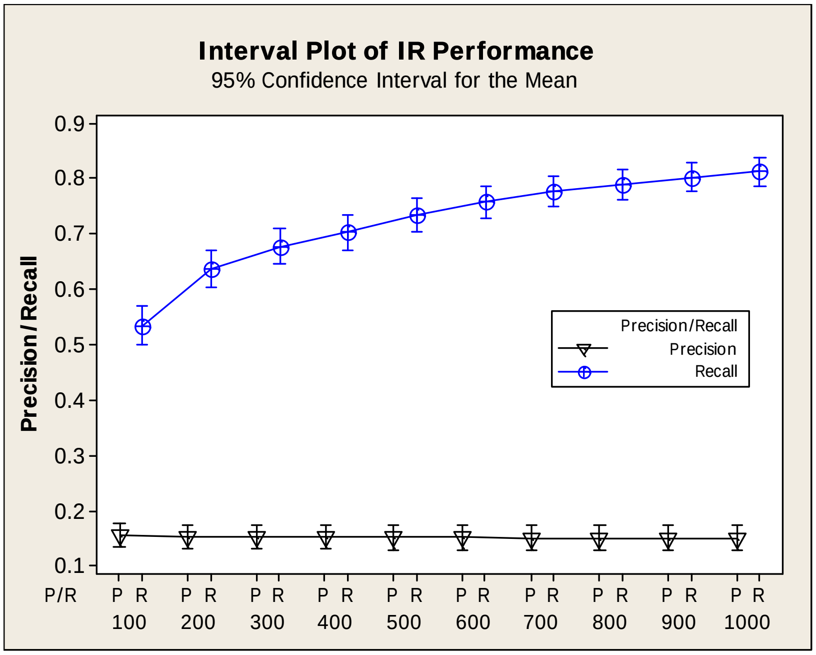

The interval plot consists of mean recall symbols with 95% confidence interval bars. Here, the two performance methods are calculated as: $Precison = \#(Relevant \cap Retrieved)/\#Retreieved$, $Recall = \#(Relevant \cap Retrieved)/\#Relevant$. We use PubMed abstracts contained in the "references" field of a protein entry from the UniProt Knowledge to estimate whether a document is relevant ("True") to a protein or not. The *x*-axis (n) indicates the maximal number of retrieved abstracts for each protein in AD-related network. For a specific n, the interval plot shows the distribution of precision and recall performance of proteins for which top n abstracts are returned from our system.

We examined the recall performance of our system by querying against a locally indexed PubMed database of 16,120,074 abstracts (2007 release). Up to top 1000 abstracts were retrieved for each of the 560 AD query proteins, resulting in a collection of 222,609 unique PubMed abstracts—4 times the size of PubMed abstracts that were retrieved with the simple query term "Alzheimer" against the same database. In this step, we were mainly interested in high recall—retrieving as many abstracts as the text mining system can reasonably handle—and precision was not an immediate concern until after we sifted through the retrieved AD abstract corpus for relevant drug information. Therefore, the choice of selecting up to 1000 PubMed abstracts per query protein was made based on a balance between computational efficiency and decent recall performance. As shown in Figure S1, retrieving only the top 100 abstracts per protein query would result in 15.5% precision and 53% recall ("true" articles were defined according to a protein's UniProt Knowledgebase protein reference field), where retrieving the top N (100<N<=1000) abstracts per protein resulted in steadily increased recall performance, up to 81% at N=1000 while keeping precision at the consistent level of around 15%. Even though N>1000 would likely further increase recall, for the purpose of computational efficiency, we chose to limit N=1000 for this study.

Method for PubMed Abstract Retrieval

An expanded list {p_1, p_2,…, p_m} containing all the proteins in a network is built as the initial query to the disease-specific biomedical literature mining system in our earlier work [1]. The system maintains a complete PubMed abstract dataset with 16 million records indexed locally, and a comprehensive protein/gene list of known name variants including acronyms, homonyms and synonyms. It automatically generates an XML query statement with directives for synonyms and additional synonyms, using searching rules from the network related protein list based on our in-house protein name thesauri database. The following is an example query statement for a sample query gene/protein *APP*:

```
<query>#syn(#uw6(amyloid beta A4 protein precursor) APP ABPP #uw5(alzheimer
disease amyloid protein) #uw5(cerebral vascular amyloid peptide) CVAP
#uw4(protease nexin II) #uw3(PN II) PreA4)</query>
```

Here, the #syn directive instructs the query analyzer to treat term or term expressions in the parameter set as synonyms, whereas the #uwN directive instructs the query analyzer to match all term expressions where the component terms are found within a neighborhood of N adjacent words in any order [2]. For example, the document (PMID=8239320) titled "regulation and expression of the Alzheimer's <u>beta/a4 amyloid protein precursor</u> in health, disease, and down's syndrome" will be retrieved by the above sample XML query, because "beta/a4 amyloid protein precursor" matches the "#uw6(amyloid beta A4 protein precursor)" query directive. In this study, we set N to be 1 plus the total count of terms within the directive to calculate similarity between query and abstracts.

References

1. Li J, Zhu X, Chen JY. Mining disease-specific molecular association profiles from biomedical literature: a case study; 2008; Fortaleza, Brazil. pp. pp. 1287-1291.

2. Metzler D, Croft WB (2004) Combining the Language Model and Inference Network Approaches to Retrieval. Information Processing and Management Special Issue on Bayesian Networks and Information Retrieval 40: 735-750.
