# Supplementary material for: Building Disease-Specific Drug-Protein Connectivity Maps from Molecular Interaction Networks and PubMed Abstracts
Source: PLoS Comput Biol. 2009 Jul 31;5(7):e1000450. doi: 10.1371/journal.pcbi.1000450 (PMC2709445; doi:10.1371/journal.pcbi.1000450)
Supplement: Text S2 — A Discussion of Statistical Components Used (0.09 MB PDF) [file pcbi.1000450.s006.pdf]

A Discussion of Statistical Components Used

In this supplement, we discuss the significance of three main statistical components used in this work. We share this discussion primarily because we believe that our readers may find it beneficial to use all or part of these techniques themselves in other related projects. The statistical components we will discuss are:

(1) Filtering and ranking of disease-related proteins in a disease-specific protein interaction subnetwork;

(2) Ranking of enriched drug terms from retrieved PubMed abstract collection;

(3) Associative mapping between proteins and drugs based on significant term co-occurrence.

Note that the first two components are used to build the two dimensions for the connectivity map separately and the third component is used to build the content of the connectivity map. The coverage of the connectivity is primarily determined with the selection of the first two components, while the quality of the final map depends on the sensitivity and specificity of both proteins and drugs selected for the connectivity map in addition to the sensitivity/specificity of protein-drug association values in the map itself.

Filtering and ranking of disease-related proteins in a disease-specific protein interaction subnetwork

The essence of this component is to use biological prior knowledge of protein interaction networks to help improve the recall of disease-related proteins. Three factors affects the performance this component: seed protein selection, protein interaction data quality and coverage, and network-based protein-ranking strategy. Based on a recent follow-up study of [1], which we just reported [2], we share the following experience with readers who may consider applying this component to improve recall of disease-relevant proteins in other projects. First, the initial quality of seeds should be based on prior curated knowledge as much possible, with Omics results performing better than text mining results. Second, disease gene ranking should be performed using PPI data with reasonable quality but as high data coverage as possible. Third, the ranking algorithm that takes advantage of local network parameters should be chosen over those using global network parameters. When high-quality seed proteins, high-quality/high-coverage PPI data, and local network parameters such as local node degrees are chosen, the sensitivity and positive predictive values of top ranked proteins from this component can be substantially improved.

Ranking of enriched drug terms from retrieved PubMed abstract collection

The essence of this component is to use false discovery rate (FDR) to identify disease-specific drugs with balanced identification sensitivity and specificity. A *p-value* is first calculated for each drug to indicate how significant each drug term is enriched in the retrieved subset against the whole PubMed abstract collection. This calculation is based on an improved term frequency based t-statistic instead of the standard approximation method (t*f*-id*f*), as a result of our study performed earlier in [3]. The reason is that use of a single term frequency in the t*f*-id*f* method for test statistic could lead to statistical test bias, especially for terms that occur rarely. Therefore, the use of term sampling technique to establish term frequency distribution for each drug terms results in improved estimation of true *p-value*

for each term. The multiple testing correction of *p-value* to FDR was a standard practice to control false positives among large number of terms determined to be significantly enriched. Our earlier study [4] also showed that FDR can be used to select the enriched drug terms from 16 subcategories of "*Chemical and Drugs*" in MeSH (Medical Subject Headings). By varying the FDR threshold, the users can control how much tolerance for "potentially novel" disease-associated drugs (and therefore potentially more false positive drugs) to allow in the final connectivity map. We chose FDR<0.05 for this work, but we believe users can also experiment with higher FDR filters, as long as the PPV can be controlled at a satisfactory level.

Associative mapping between proteins and drugs based on significant term co-occurrence.

The essence of this component is to use log-odds ratio to quantify connectivity strengths for each possible pair of proteins and drugs. The calculation of connectivity score Θ can be inferred as:

$$\Theta_{pd} = \ln(df_{pd} * N + \lambda) - \ln(df_p * df_d + \lambda)$$

$$= \ln\left(\frac{df_{pd} * N + \lambda}{df_p * df_d + \lambda}\right) \approx \ln\left(\frac{df_{pd} * N}{df_p * df_d}\right) = \ln\left(\frac{P(p,d)}{P(p) * P(d)}\right) = \ln\left(\frac{P(p \mid d)}{P(p)}\right) = \ln\left(\frac{P(d \mid p)}{P(d)}\right)$$

Where, *P(p,d)* is the co-occurrence probability of protein *p* and drug *d. P(p)* and *P(d)* are the occurrence probability protein *p* and drug *d* in the entire data set respectively. *P(p|d)* is the occurrence probability of protein *p* in the contexts of drug *d*. *P(d|p)* is the occurrence probability of drug *d* in the contexts of protein *p*. The connectivity score Θ_{pd} >0, when the protein-drug pair is over-represented (*i.e., P(d|p)>p(d), or P(d|p)>p(d)).*

Therefore, the more often that two terms (one for protein and the other for drug) are co-cited together, the higher the final score. However, this connectivity score should not be confused with the broad, untargeted "fishing" for protein-drug terms from the whole PubMed, because only disease-specific PubMed subset enriched with molecular study information is used. In this study, we have shown good sensitivity and accuracy of such profiles, when clustered based on drugs' connectivity profile, in determining structure similarity for some clustered drugs. However, we believe the future incorporation of semantics information of the co-cited text may improve the accuracy of the connectivity map.

References:

1. Chen JY, Shen C, Sivachenko A (2006) Mining Alzheimer Disease Relevant Proteins from Integrated Protein Interactome Data. Pacific Symposium on Biocomputing '06. Maui, HI. pp. 367-378.
2. Huang H, Li J, Chen JY. Disease Gene-fishing in Molecular Interaction Networks: a Case Study in Colorectal Cancer 2009; Minneapolis, MN.
3. Li H, Chen JY (2009) Improved biomedical document retrieval system with PubMed term statistics and expansions. International Journal of Computational Intelligence in Bioinformatics and Systems Biology 1: 74-85.
4. Li J, Zhu X, Chen JY (2009) Discovering Breast Cancer Drug Candidates from Biomedical Literature. International Journal of Data Mining and Bioinformatics.
